# A case of dystrophic epidermolysis bullosa with a rare COL7A1 variant

**DOI:** 10.1016/j.abd.2022.09.020

**Published:** 2024-02-19

**Authors:** Patrícia Amoedo, Ana Grangeia, Lígia Peralta, Alberto Mota

**Affiliations:** a Dermatology and Venereology Service, Centro Hospitalar Universitário de São João, Porto, Portugal; b Medical Genetics Service, Centro Hospitalar Universitário de São João, Porto, Portugal; c Neonatology Service, Centro Hospitalar Universitário de São João, Porto, Portugal; d Facudade de Medicina da Universidade do Porto, Porto, Portugal; e Centro de Investigação em Tecnologias e Serviços de Saúde, Porto, Portugal

*Dear Editor,*

A full-term male infant was born with bullae and several eroded areas, mainly in the feet and the face, but also affecting the trunk, scalp, and perineal region (Figure 1, Figure 2). He also presented corneal opacity in the right eye with hypervascularization and hemorrhage. Nails, hair, and mucosae were normal. The patient was the first child of a healthy, non-consanguineous couple with no family history of blistering skin diseases. A 22 gene panel performed by Next-Generation Sequencing (NGS) found a pathogenic variant, c.325_326insCG (p.Glu109Alafs*39), in homozygosity in the COL7A1 gene. In the first months of life, the condition worsened with new lesions on the hands and armpits and mutilating scarring of the feet. Unfortunately, parents declined further studies. Based on clinical presentation and genetic analysis, a diagnosis of Severe Recessive Dystrophic Epidermolysis Bullosa was made.

**Figure 1:**
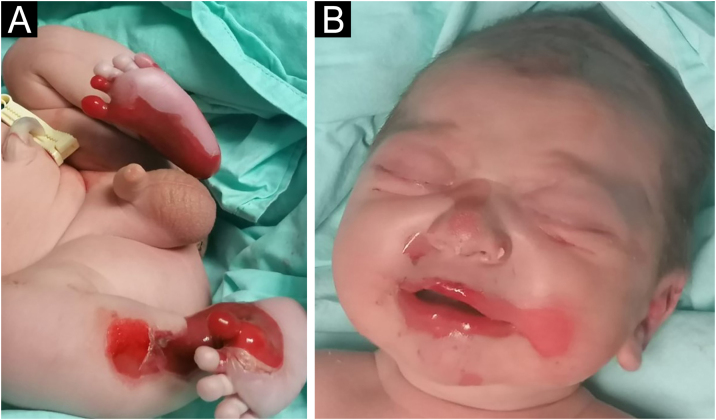
Extensive areas of skin detachment on the anteromedial aspects of both lower extremities (A) and perioral region (B).

**Figure 2:**
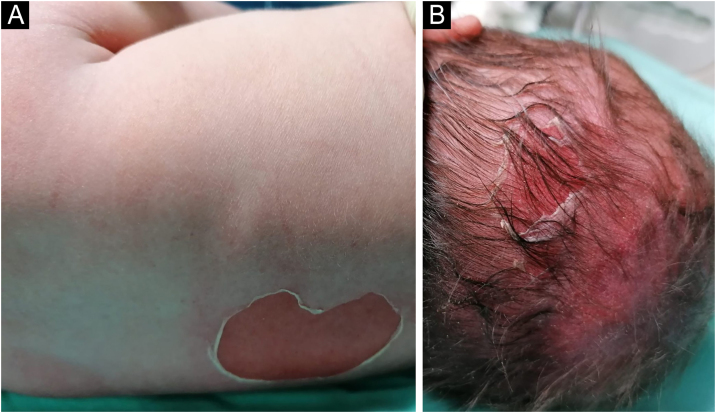
Blistering and skin detachment at trunk (A) and scalp (B).

Dystrophic Epidermolysis Bullosa (DEB) is an inherited skin fragility disorder, characterized by blister formation in the sublamina densa. It is caused by pathogenic variants in the COL7A1 gene, that encodes type VII collagen, responsible for a cohesive dermal-epidermal junction. The inheritance pattern could be Recessive (RDEB) or Dominant (DDEB).^1^, ^2^ The clinical spectrum is highly variable, from generalized blistering forms, with mucous involvement, to mild localized ones, and, in general, recessive forms are more severe.^2^ The latter is usually associated with variants causing Premature Stop Codons (PTC), with subsequent decay or truncated polypeptides. In heterozygosity, PTC variants do not cause disease, but compound heterozygosity with missense variants could be associated with milder forms of RDEB. DDEB is generally caused by glycine substitutions, but other mutations could be involved. Glycine substitutions could also be inherited recessively, and some specific substitutions were even associated with both RDEB and DDEB.^1^, ^2^, ^3^

The severity of symptoms depends on the level of COL7A1 expression, which is determined by the type and position of the pathogenic variant.^1^, ^2^, ^3^ However, genotype-phenotype correlation is not consistent and identical variants could result in different phenotypes, which suggests that other factors, genetic or environmental, may be involved in this phenotype divergence.^1^, ^4^

The variant presented in our patient is rare, with less than 10 cases previously reported. Interestingly, this variant was only reported in northern parts of Portugal and Brazil, which could be due to a founding effect, related to the former Portuguese settlement.^1^

The present case contributes to the mutational spectrum associated with RDEB and to a better understanding of its clinical correlation.

## Financial support

None declared.

## Authors’ contributions

Patrícia Amoedo: Writing and editing (lead).

Ana Grangeia: Review (support).

Lígia Peralta: Review (support).

Alberto Mota: Review and final approval.

## Conflicts of interest

None declared.
